# New insights into the molecular mechanism behind mannitol and erythritol fructosylation by β-fructofuranosidase from *Schwanniomyces occidentalis*

**DOI:** 10.1038/s41598-021-86568-6

**Published:** 2021-03-30

**Authors:** David Rodrigo-Frutos, Elena Jiménez-Ortega, David Piedrabuena, Mercedes Ramírez-Escudero, Noa Míguez, Francisco J. Plou, Julia Sanz-Aparicio, María Fernández-Lobato

**Affiliations:** 1 Centro de Biología Molecular Severo Ochoa (CBMSO; UAM-CSIC), Departamento de Biología Molecular, Facultad de Ciencias, Universidad Autónoma de Madrid, Nicolás Cabrera 1, 28049 Madrid, Spain; 2 Departamento de Cristalografía y Biología Estructural, Instituto de Física-Química Rocasolano (CSIC), Serrano 119, 28006 Madrid, Spain; 3 Instituto de Catálisis y Petroleoquímica (ICP-CSIC), Marie Curie 2, 28049 Madrid, Spain

**Keywords:** Biochemistry, Biotechnology, Structural biology

## Abstract

The β-fructofuranosidase from *Schwanniomyces occidentalis* (Ffase) is a useful biotechnological tool for the fructosylation of different acceptors to produce fructooligosaccharides (FOS) and fructo-conjugates. In this work, the structural determinants of Ffase involved in the transfructosylating reaction of the alditols mannitol and erythritol have been studied in detail. Complexes with fructosyl-erythritol or sucrose were analyzed by crystallography and the effect of mutational changes in positions Gln-176, Gln-228, and Asn-254 studied to explore their role in modulating this biocatalytic process. Interestingly, N254T variant enhanced the wild-type protein production of fructosyl-erythritol and FOS by $\sim$ 30% and 48%, respectively. Moreover, it produced neokestose, which represented $\sim$ 27% of total FOS, and yielded 31.8 g l^−1^ blastose by using glucose as exclusive fructosyl-acceptor. Noteworthy, N254D and Q176E replacements turned the specificity of Ffase transferase activity towards the synthesis of the fructosylated polyols at the expense of FOS production, but without increasing the total reaction efficiency. The results presented here highlight the relevance of the pair Gln-228/Asn-254 for Ffase donor-sucrose binding and opens new windows of opportunity for optimizing the generation of fructosyl-derivatives by this enzyme enhancing its biotechnological applicability.

## Introduction

The β-fructofuranosidase (EC 3.2.1.26) from the ascomycetous yeast *Schwanniomyces occidentalis* (Ffase) is a robust glycoside hydrolase (GH) that releases fructose units from sucrose and inulin (hydrolytic activity) but also produced 6-kestose, 1-kestose, neokestose (all trisaccharides) and blastose (disaccharide) by transfructosylation of sucrose (transferase activity; see Supplementary Fig. S1 online)^1,2^. 6-Kestose and 1-kestose belong, respectively, to the levan- and inulin-type of fructooligosaccharides (FOS), and contain fructose units linked by β-(2 → 6) (^6^F-FOS series) or β-(2 → 1) (^1^F-FOS series) bonds. Neokestose and blastose are neolevan-type sugars with fructose units linked by a β-(2 → 6) bond to the glucose moiety (^6^G-FOS series)^1–4^.

The study of the 3D structure of the enzyme revealed important insights into the mechanism underlying its hydrolase/transferase capability^4,5^. Ffase is a butterfly-shaped homodimer of 160 KDa included in the GH family 32 (GH32). Each subunit of 535 amino acids presents a bimodular arrangement with a N-terminal five fold β-propeller catalytic domain and a C-terminal β-sandwich supplementary domain linked by a short residue segment. The active site pocket of each monomer presents up to five binding subsites to the substrate and is unusually surrounded by loops from the β-sandwich domain of the adjacent subunit, which seems to contribute to allocating distal parts of oligofructans. As with other β-fructofuranosides of the same family, Ffase performs its enzymatic action through a pair of key negatively-charged amino acids in its active site, namely, Asp-50 acting as a nucleophile and Glu-230 as an acid/base catalyst. The tendency of this enzyme to produce β-(2 → 6)-linked FOS was related to the presence of several unique polar residues near to Glu-230^4,5^. Among them, the pair Gln-228/Asn-254, structurally equivalent to the Asp-239/Lys-242 pair of plant invertases^5^, was demonstrated by targeted mutation analysis to be significantly involved in the configuration of the acceptor-substrate binding site for transfructosylation and in the modulation of the enzymatic transfer specificity. Moreover, the Gln-176 residue linked to Arg-178 from the RDP motif, a specific Arg-Asp-Pro triad sequence evolutionarily preserved in β-fructofuranosidases and fructosyl-transferases for recognizing of the fructopyranosidic ring and stabilizing the transition state^6,7^, has proven to have a moderate influence on the substrate specificity and the catalytic efficiency of Ffase^5^. In addition to this, Ffase was found to have high fructosyl-acceptor promiscuity, being able to fructosylate 17 different hydroxylated compounds including small sugars and alditols, to produce new fructo-conjugates. Best alternatives to sucrose acceptor were mannitol and erythritol that resulted in the formation of 1-O-β-D-fructofuranosyl-D-mannitol and 1/4-O-β-D-fructofuranosyl-D-erythritol^1^.

Alditols are a set of aliphatic polyhydric alcohols formally arising from the reduction of the carbonyl group of monosaccharides^8^. To a varying degree, these polyols are water-soluble, hardly-crystallizable, non-cariogenic, and poorly metabolizable, while offering a sweet taste and a cooling effect. Alditols are extensively used in numerous pharmaceutical, food, and domestic products usually as sugar substitute, humectant, thickening, or plasticizer agent. Furthermore, these compounds are also widely employed as mild laxative, diuretic, hypotensive, or cryoprotectant^8–14^. In general, alditols are partially absorbed by passive diffusion in the small intestine, being excreted intact in the urine or metabolized to an extent in the liver, with some portion reaching the colon where it can be either substrate for fermentation or directly discharged in the feces^9,15,16^. Interestingly, certain polyols were shown to increase the number of bifidobacteria or lactobacilli in the human gut microbiota pointing to their use as potential prebiotics^17–19^. In this context, the transglycosylation of polyols has been previously applied to improve their functional properties (such as solubility or flavor) or to diminish their absorption to reach the large intestine. Thus, the maltosylation of erythritol enhanced its sweetness without affecting its physical–chemical properties^20^, the glucosylation of mannitol increased its solubility in water^21^, and the galactosylation of sorbitol raised the microbiotic population of *Bifidobacterium, Lactobacillus* and *Streptococcus* species^22^.

Some microbial enzymes have been reported to produce glycosyl-derivatives from alditols such as the levansucrases SacB from *Bacillus subtillis*^23^ and Lsc3 from *Pseudomonas syringae*^24^, the α-glucosidase HaG from *Halomonas sp.* H11^25^, the β-glucosidases CelB from *Pyrococcus furiosus*^26^ and SSG from *Sulfolobus shibatae*^27^, the β-galactosidases Bgal from *Kluyveromyces lactis*^28^ and LacA from *Aspergillus oryzae*^29^, or the β-N-acetylhexosaminidase from *Bacillus sp.* CH11^30^. However, to the best of our knowledge, there are no known β-fructofuranosidases described to generate such compounds other than that from Arthrobacter sp. K-1 (renamed to *Micobacterium saccharophilum* K-1)^31,32^ and Ffase^1^, which undoubtedly increases the biotechnological potential of this enzyme. Both, the *M. saccharophillum* K-1 enzyme and levansucrases are GH68 enzymes sharing catalytic machinery with GH32 within GH-J clan (ww.cazy.org), but differing in their transferase specificity.

This work aims to evaluate the structural determinants of Ffase ruling the transfructosylation process of the alditols mannitol and erythritol, so that a greater understanding of this peculiar biocatalytic activity can be achieved. This could thereby facilitate the creation of a cost-effective method for producing fructosyl alditols.

## Results

### Structural analyses of Ffase with polyols as fructosyl-acceptors

**Figure 1:**
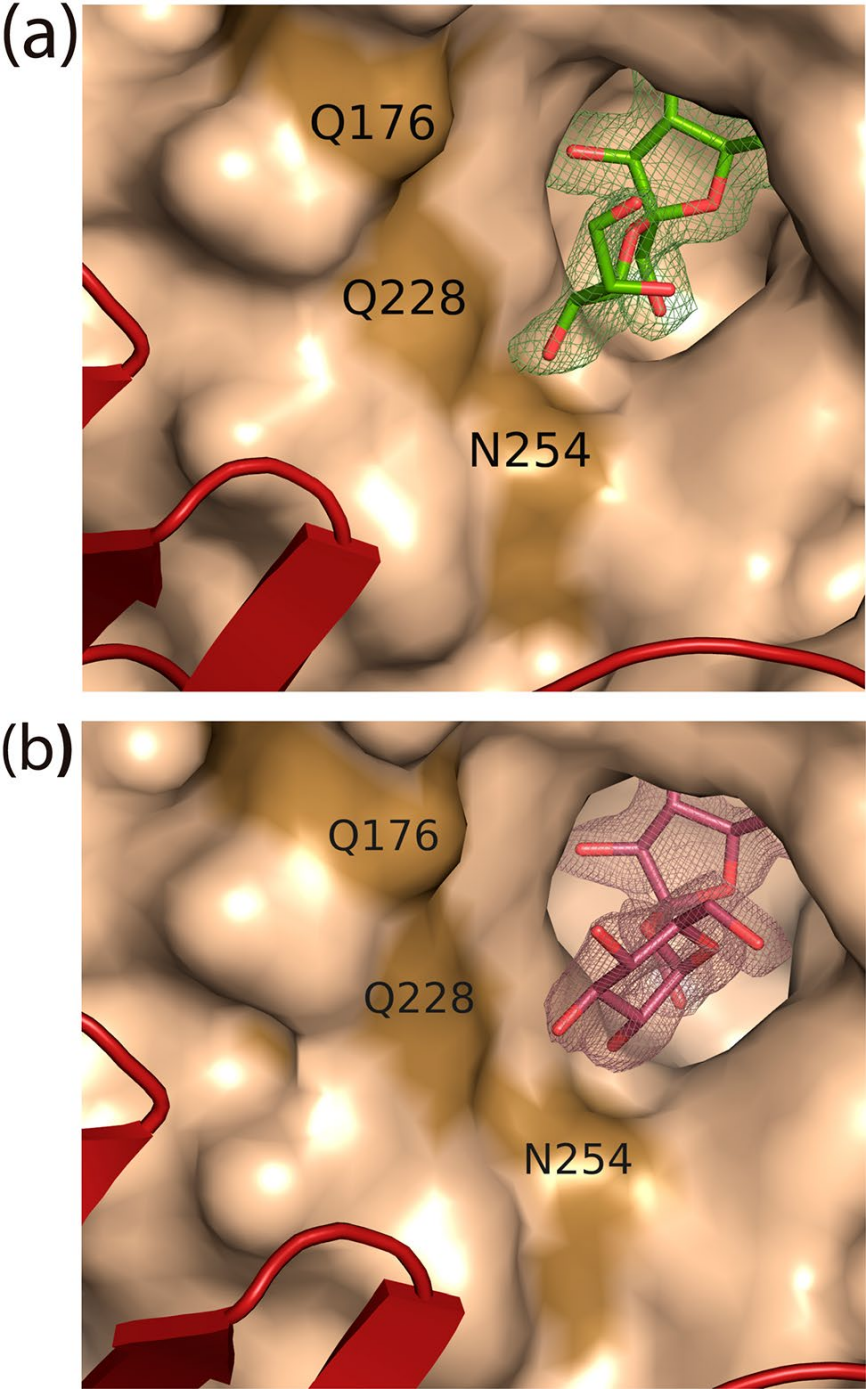
Surface representation of a Ffase-D50A subunit showing the active site complexed with (**a**) fructosyl-erythritol (green) or (**b**) sucrose (prune). The 2FoFc electron density maps are contoured at 1σ. A small part of the second subunit is shown as red cartoon.

As explained before, the crystallographic study of the Ffase homodimer and its subsequent evaluation by site-directed mutagenesis disclosed the key determinants for its transfructosylating activity with sucrose. The pair Gln-228/Asn-254, and to a lesser extent Gln-176/Arg-178, was found to have an important role for regulating the biocatalytic capacity of this enzyme as well as controlling its specificity for FOS production^4,5^. In this context, further structural research was undertaken to understand the ability of Ffase to use sucrose-alternative compounds as fructosyl-acceptors and generate derivatives from the alditols mannitol and erythritol^1^. To this aim, the inactivated mutant Ffase-D50A was expressed in *Saccharomyces cerevisiae*, purified, and crystallized as previously referred^4^. The resultant protein crystals were then soaked within β-fructose plus either mannitol or erythritol to obtain the corresponding intermediate complex with these two molecules. Unfortunately, only fructose was bound at the active site in both experiments. Thereby, the enzyme was directly incubated with purified fructosyl-mannitol or fructosyl-erythritol to obtain the corresponding complex, and was submitted to crystallization experiments. Data collection and structure determination of these crystals are shown in Table S1. The formation of Ffase-D50A/fructosyl-erythritol complex was clearly detected (Fig. 1a). However, only sucrose was observed in crystals grown in the presence of fructosyl-mannitol (Fig. 1b), most probably due to traces of this disaccharide in the purified product. Experimental details of the crystal structure determination of both complexes are given in Table S1.

**Figure 2:**
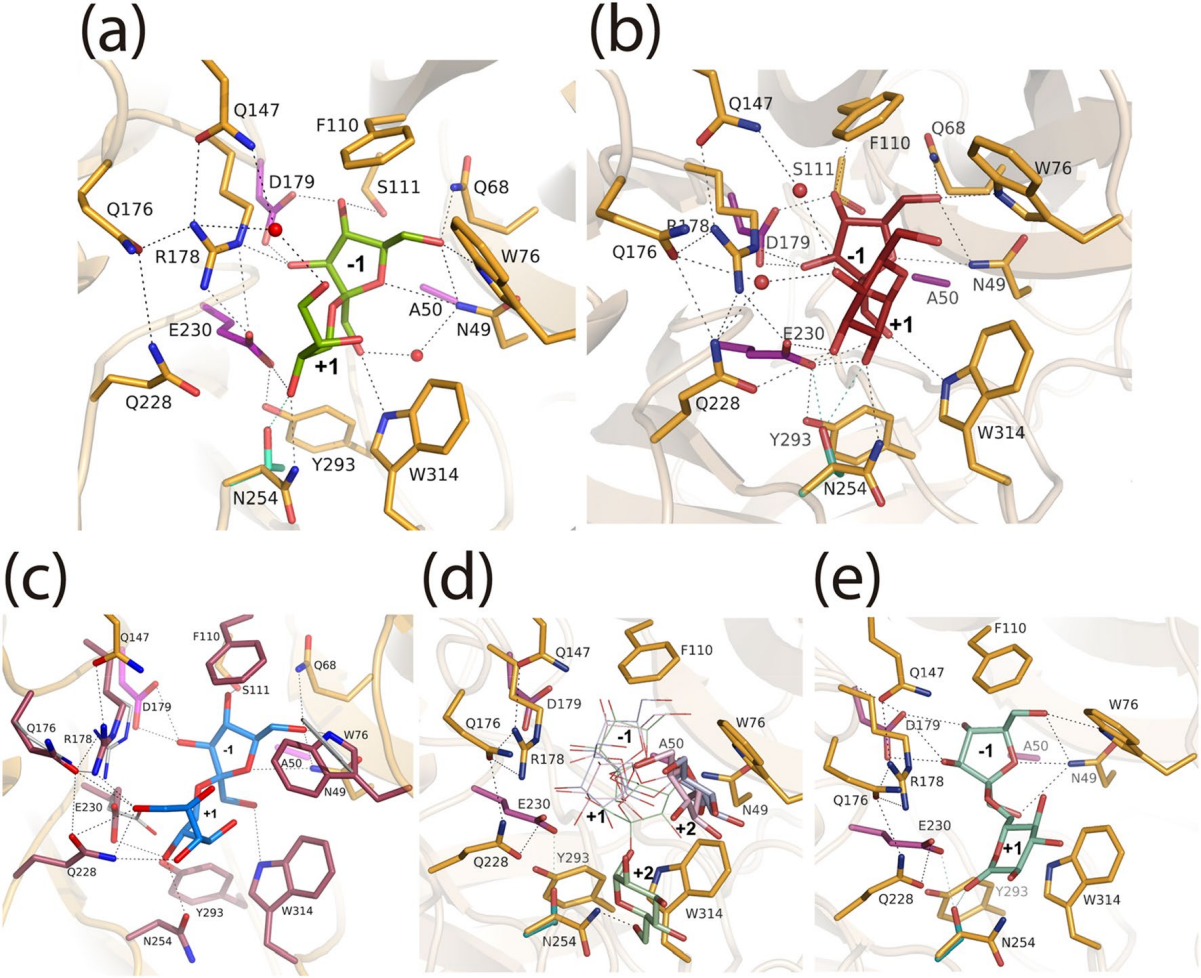
Detailed image of the atomic interactions produced at the Ffase active site. Crystallographic complexes with (**a**) fructosyl-erythritol (green) or (**b**) sucrose (prune) are shown, with relevant residues represented as orange sticks, and the catalysts in magenta. Polar interactions are represented as dashed lines. The modelled Thr in the N254T mutant is represented in cyan, with its putative interactions in the same color. (**c**) Docking simulation of the fructosyl-mannitol derivative (marine) into the Ffase active site, as computed by AutoDock Vina; the residues considered as flexible in the calculation are colored in raspberry, showing in grey sticks the corresponding position observed in the fructosyl-erythritol of those that have moved in the model. (**d**) The modelled position of the products 6-kestose (pale green), 1-kestose (pale blue), and neokestose (pink), reported previously^2^, is shown with the terminal sugars located at subsite + 2 highlighted as broader sticks. (**e**) The modelled position of blastose showing its putative interaction with Thr in the mutant N254T.

Figure 2a shows a detail of the fructosyl-erythritol recognition pattern within the Ffase active center. The fructo-conjugate is spanning subsites − 1 and + 1 in the expected product binding mode. On the one hand, the fructose bound at subsite − 1 is fixed by many direct polar interactions of all its hydroxyl groups and the ring oxygen to Asn-49, Gln-68, Trp-76, Ser-111, Arg-178, and Asp-179 amino acid side-chains. A water molecule is occupying a position close to the missing nucleophile carboxylate acting as a link between O1 of fructose and the Ala-50 NH main-chain. This binding mode is highly conserved within the GH32 enzymatic family and determines its specificity for fructo-based substrates. On the other hand, the erythritol moiety is accommodated by polar interactions between the secondary hydroxyl group next to the fructosyl linkage and the catalytic Glu-230 and to Asn-254 side-chains. Furthermore, as it is described in more detail below, the terminal primary hydroxyl is linked to Gln-147 through a well-ordered water molecule that participates in a network connecting residues in the surroundings of the catalytic site.

Figure 2b illustrates in detail the binding mode of the sucrose donor substrate. The fructose located at − 1 presents the same atomic interactions described above and conserved within the GH32 family. For its part, the glucose at + 1 is recognized through a complex net of direct and water-mediated polar linkages. First, there is a bifurcated hydrogen bond between both oxygens from the catalytic Glu-230 carboxylate to the O2 and O3 of glucose, these atoms in turn being also linked to Gln-228 and Asn-254 side-chains, respectively. Moreover, the glucose O4 is linked through two water molecules to Gln-147, Gln-176, and Gln-228. Consequently, the pair Gln-228/Asn-254, previously defined as a hotspot for regulating the transfructosylated product specificity, is also directly involved in the donor-sucrose binding, which necessarily must influence the first step of the enzymatic mechanism with alternative acceptors. Moreover, the Ffase-sucrose complex shows several long-chain residues in the environment of the catalytic Glu-230 that make a complicated network of polar linkages, keeping a tight architecture at this part of the catalytic site. Thus, in addition to Arg-178 at the conserved RPD motif, Gln-176 and Gln-228 are making an intricate polar net for keeping Glu-230 side-chain in a very precise conformation and for stabilizing the binding of sucrose.

As the soaking experiment to obtain Ffase-D50A/fructosyl-mannitol complex failed, a docking simulation of the fructosylated derivative into the Ffase active site was performed using AutoDock Vina. From the 20 calculated conformers, the first energy-ranked solution presented the more conservative binding of the product at subsite − 1, as compared to the Ffase-D50A crystallographic complexes with fructosyl-erythritol and sucrose here reported. As it is shown in Fig. 2c, fructose keeps essentially the same polar interactions at its hydroxyls with the residues Asn-49, Gln-68, Trp-76, Ser-111, Asp-179 and Trp-314 at the Ffase active site, therefore supporting the calculated complex model. Furthermore, an inspection to the figure shows that mannitol is tightly fixed at subsite + 2 by many polar interactions of its O2 to the Gln-228 and Ans-254 side-chains, its O4 and O6 to Gln-288, the O6 being additionally linked to Gln-176 and Arg-178 in the complex net of polar interactions linking Glu-230 to Arg-178 and Gln-176, described above. It should be noted that some conformational changes are observed in the modelled positions of these residues, which highlights their putative role in accommodating mannitol.

On the basis of these structural features, the catalytic effect induced by changes in positions Gln-176, Gln-228, or Asn-254 on the production of fructosylated alditols was revised. The final goal was to explore the potential use of the variants to enhance the biotechnological applicability of Ffase.

### Assessment of Ffase N254 variants for fructosyl-erythritol synthesis

Native Ffase directly purified from *Sw. occidentalis* and incubated at 50ºC for 2 h in a reaction mixture containing 200 g l^−1^ sucrose and 500 g l^−1^ erythritol produced 35 g l^−1^ of 1/4-O-β-D-fructosyl-erythritol, this derivative being the major transfer product and accounting for around 5% (w/w) of all compounds thereof^1^. Considering the major role of Asn-254 in binding the erythritol moiety of the fructosyl-erythritol (Fig. 2a), the production of this compound was evaluated using four different protein variants including substitutions N254D/T/A/E, which had previously been expressed in *S. cerevisiae* and showed capacity to produced FOS, mainly 6-kestose from sucrose^4^. Recombinant proteins were first purified and then incubated in reaction mixtures under the same experimental conditions already mentioned that were concomitantly followed by HPLC-ELSD. As expected, clear peaks corresponding to fructosyl-erythritol were detected in all chromatograms analyzed, which constituted the major product synthesized by the tested variants. Supplementary Fig. S2 shows a couple of representative chromatograms and Table 1 summarizes the level of transfructosylated products at the reaction time in which a maximum amount of fructosyl-erythritol is obtained. After 3 h, wild-type Ffase produced 37.2 g l^−1^ of fructosyl-erythritol (5.3% of the total amount of all compounds in the reaction mixture) and 3.7 g l^−1^ of FOS (of which 62% was 6-kestose).

**Table 1:** Concentration (g l^−1^) of the sugar constituents present at maximum fructosyl-erythritol production in reaction mixtures based on sucrose and erythritol with the referred Ffase variants.

Ffase	Sucrose	F-erythritol	Neokestose	1-Kestose	6-Kestose	FOS^†^	Ratio^‡^
*wt*	92.3 ± 4.6	37.2 ± 2.0	0.58 ± 0.02	0.82 ± 0.03	2.3 ± 0.1	3.7	10
N254D	77.6 ± 3.9	35.4 ± 1.6	ND	0.27 ± 0.01	0.83 ± 0.04	1.1	32
N254E	168.4 ± 8.2	10.5 ± 0.5	ND	ND	ND	ND	–
N254T	97.7 ± 4.5	48.8 ± 2.3	1.52 ± 0.08	0.98 ± 0.04	3.0 ± 0.1	5.5	9
N254A	175.7 ± 9.1	6.6 ± 0.3	ND	ND	ND	ND	–

^†^Concentration of FOS: neokestose, 1-kestose and 6-kestose.

^‡^ Ratio between fructosyl-erythritol (F-erythritol) and total FOS (weight ratio).

ND: Not detected.

Data represents the average of two independent measurements obtained after 3 h and 2 h of reaction for variants *wt*/N254E/A and N254D/T, respectively. Standard deviation values are indicated.

**Figure 3:**
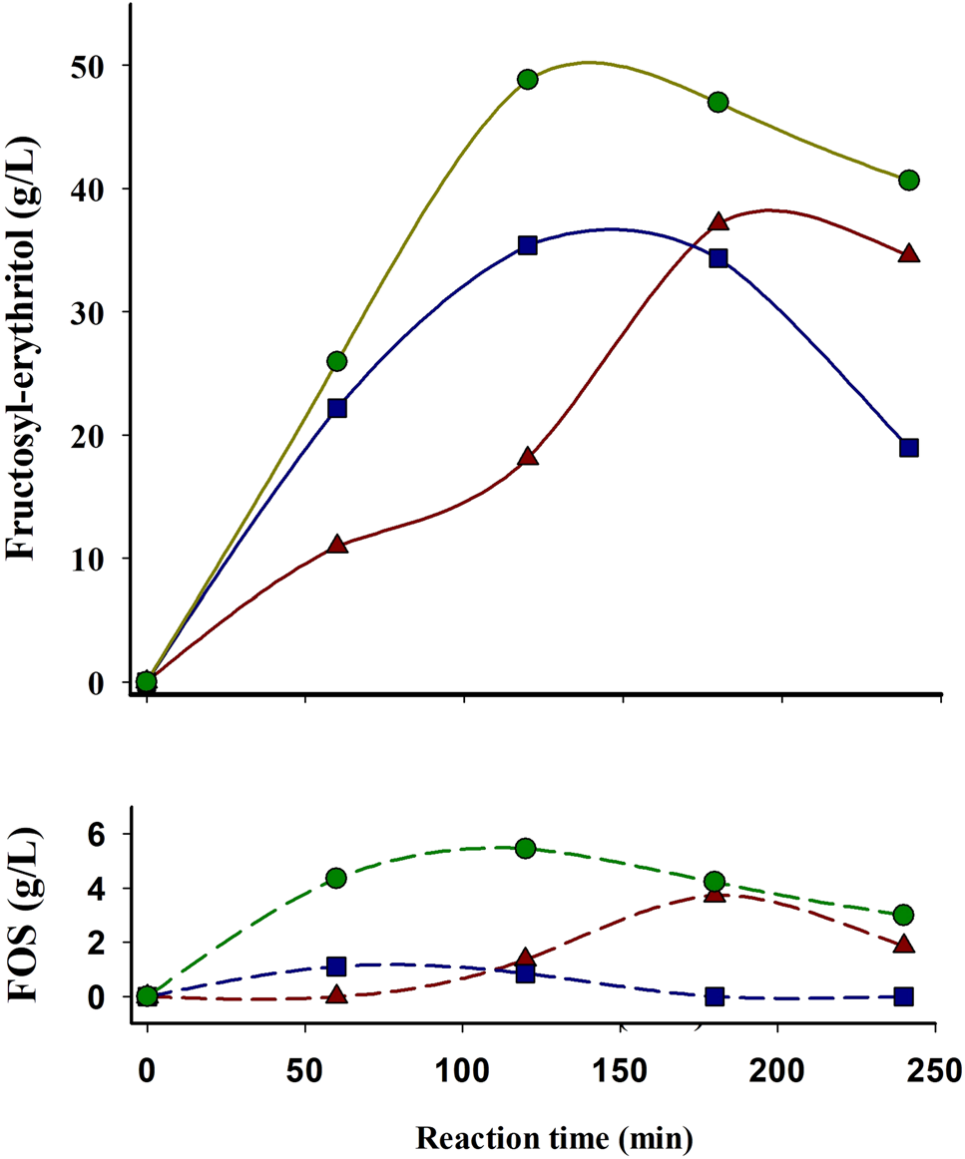
Time course of fructosyl-erythritol and total FOS production by the wild-type Ffase and the indicated variants. Reaction mixtures (1.5 ml) incubated at 50 ºC contained 10 U ml^−1^ of the referred enzymatic variant (previously purified), 200 mg ml^−1^ sucrose, and 500 mg ml^−1^ erythritol in 0.1 M sodium acetate pH 5.5. The constituents of the reaction mixtures were quantified in duplicate at the indicated times by HPLC-ELSD with standard deviation less than 5%. Data obtained with the wild-type (brown triangles), N254T (green circles) and N254D (blue squares) variants are presented.

Enzymatic variants including substitutions N254E and N254A showed very small hydrolase and transferase activity, both producing 10.5 and 6.6 g l^−1^ of fructosyl-erythritol, respectively, values that decay to 9.7 and 6.5 g l^−1^ after 4 h (data not shown). In addition, neither of these two variants generated any type of FOS under these conditions, highlighting the importance of residue N254 in the recognition of substrates/products and hydrolysis that we had previously described by using fructans of different chain size^4^. However, the variant Ffase-N254D basically maintained the wild-type fructosyl-erythritol production level after 2 h, but with a reduction in the total amount of FOS by almost 60%, 1.1 *versus* 3.7 g l^−1^ (Table 1 and Fig. 3), which increased the fructosyl-erythritol/FOS ratio by 3.3-fold. In contrast, the N254T mutant enhanced both, production of fructosyl-erythritol and FOS by $\sim$ 30% (48.8 g l^−1^) and 48% (5.5 g l^−1^), respectively. As previously reported when using reactions based exclusively on sucrose^4^, the N254T substitution produced a slight shift on the Ffase activity towards the production of neokestose, which in reactions including erythritol represented $\sim$ 27% of the total FOS produced *versus* the $\sim$ 16% of the wild-type enzyme.

**Figure 4:**
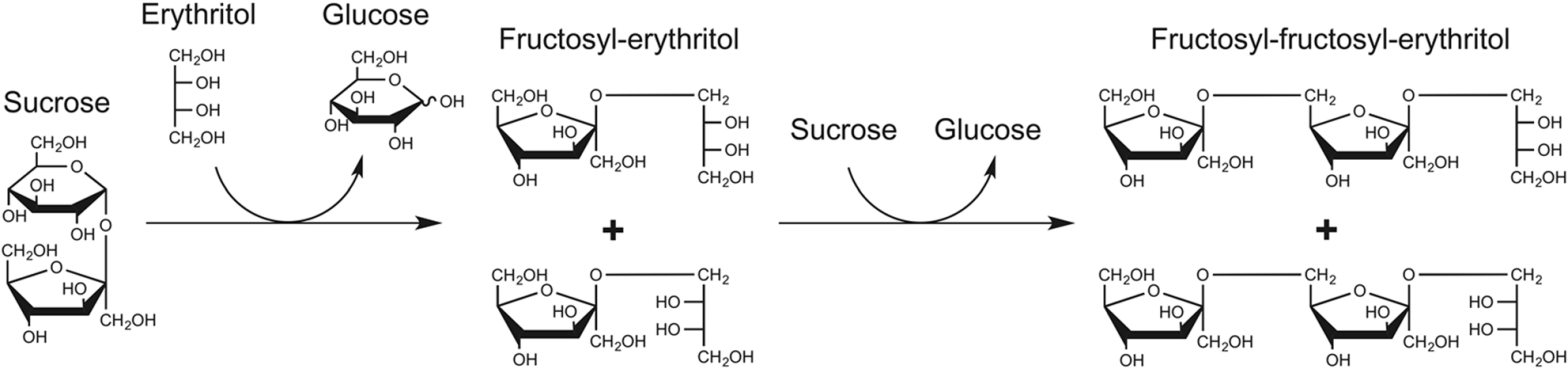
Schematic view of the reactions based on erythritol mediated by Ffase. The structure of the two fructosyl-erythritol enantiomers previously detected in the reaction^1^ are generated due to a difference in the position of fructosylation of erythritol: 1-O-β-D-fructosyl-D-erythritol (when the linkage occurred next to the chiral center R) and 4-O-β-D-fructosyl-D-erythritol (if it occurred next to the S one). Therefore, two potential structures for fructosyl-fructosyl-erythritol detected in reactions mediated by the Ffase-N254T are also possible.

In addition, in the chromatograms obtained with the N254T variant, peaks corresponding to small traces (< 0.1 g l^−1^) of the disaccharide blastose and to a new product that very probably could be fructosyl-fructosyl-erythritol were also detected (see Supplementary Fig. S2 online). In fact, analysis of the reaction mixture by MS showed a clear peak of [M + Na]^+^ m/z of 469.2 (Supplementary Fig. S3 online), which may correspond to the double fructosylation of erythritol. Unfortunately, the low generated amount of the proposed fructosyl-fructosyl-erythritol did not allow its structural characterization by nuclear magnetic resonance techniques. A schematic view of the reactions mediated by the variant Ffase-N254T with erythritol is represented in Fig. 4.

### Assessment of Ffase-N254T for blastose synthesis

**Figure 5:**
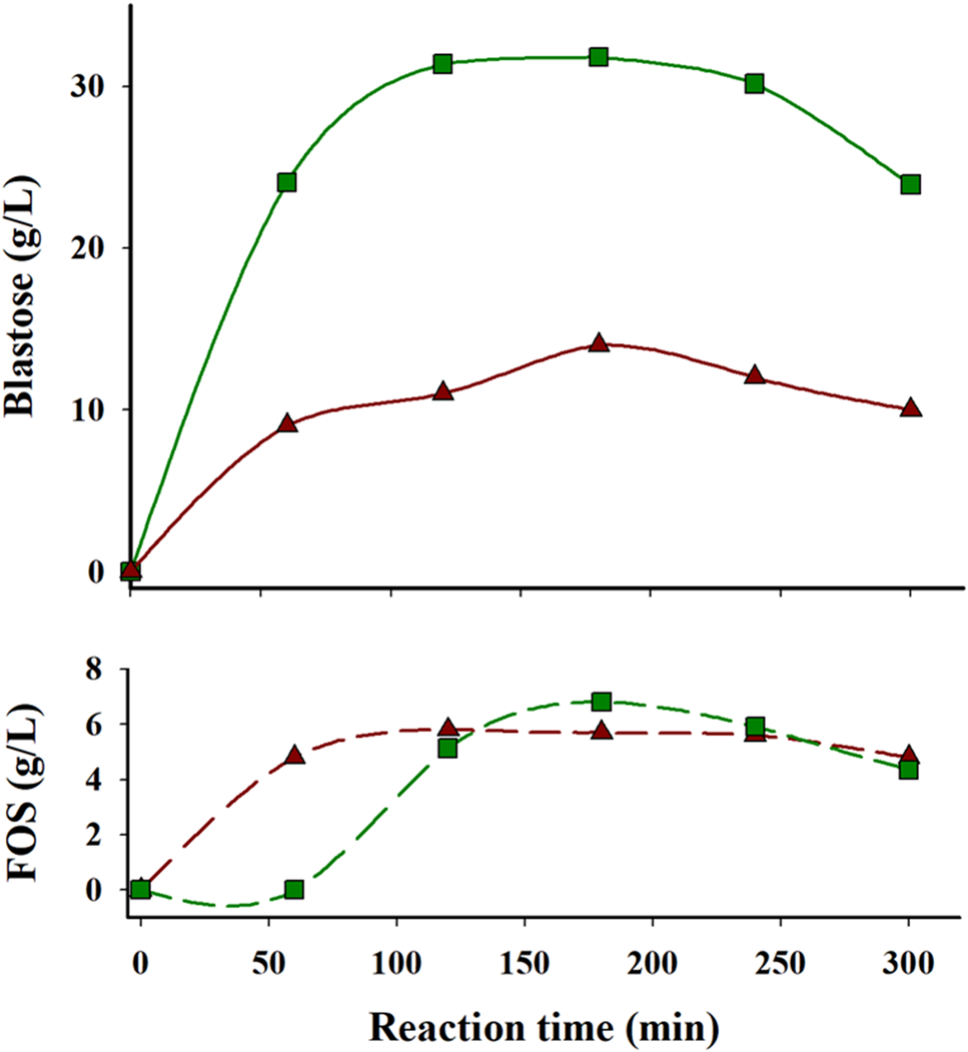
Time-course of the reaction mediated by the wild-type Ffase and N254T variant using glucose as alternative fructosyl-acceptor. Data from native Ffase and Ffase-N254T are represented in brown triangles and green squares, respectively. FOS for the wild-type enzyme were 6-kestose and 1-kestose; while for the N254T variant were neokestose and 6-kestose. Reaction mixtures (1.5 ml) incubated at 50 ºC contained 10 U ml^−1^ of the purified enzymatic variants, 200 mg ml^−1^ sucrose, and 600 mg ml^−1^ glucose in 0.1 M sodium acetate pH 5.5. The constituents of the reaction mixtures were quantified in duplicate at the indicated times by HPLC-ELSD with standard deviation less than 5%.

Native Ffase is able to fructosylate glucose to produce blastose disaccharide whose sugar units are connected by a β-(2–6)-linkage^1,2^. The relevance of position Asn-254 in the product specificity of Ffase and the shift of the Ffase-N254T variant towards production of neokestose, a neo FOS with a β-(2–6)-linkage between fructose and the glucosyl moiety of sucrose (Fig. 4), was already demonstrated^4^. In this work, the potential of the N254T variant to synthesize blastose was analyzed in detail using 200 g l^−1^ of sucrose (fructosyl donor) and 600 g l^−1^ of glucose (fructosyl acceptor). The largest amount of blastose detected in these conditions after 3 h was 31.8 g l^−1^, which represented 82.4% (w/w) of the total amount of transfructosylated products in the reaction mixture (Fig. 5) and 4% of the total compounds in the mixture. At this time, the highest concentration of FOS was found to be 3.9 g l^−1^ neokestose and 2.8 g l^−1^ 6-kestose. 1-Kestose was not detected in any of the HPLC chromatograms analyzed using Ffase-N254T variant under these conditions, which again clearly indicates that its product specificity is altered towards the formation of β-(2–6) bonds to the detriment of the β-(2–1)-linkages. By contrast, wild-type Ffase produced 14.6, 4.4, and 2.2 g l^−1^ of blastose, 6-kestose, and 1-kestose, respectively, without detection of neokestose.

### Assessment of Ffase Q176 and Q228 variants for fructosyl-mannitol synthesis

Mannitol is, as afore-mentioned, one of the best-known Ffase fructosyl-acceptors. The enzyme was previously shown to produce 43.8 g l^−1^ of fructosyl-mannitol (7.3% of the total amount of all compounds present in the mixture) after incubation with 200 g l^−1^ sucrose and 400 g l^−1^ mannitol for 1.5 h^1^. Our failure in getting crystals with this product led us to explore other considerations to identify putative residues regulating this process. Interestingly, the structural study of the Ffase/sucrose complex described here (Fig. 2b) highlighted the key role of Gln-176 and Gln-228 in fixing the position of the acid/base catalyst Glu-230 carboxylate, Gln-228 also making a direct hydrogen bond to the substrate. Moreover, the computed model of the complex calculated with AutoDock Vina points to the direct implication of these two residues in accommodating the mannitol moiety of the fructosylated product through several polar interactions (Fig. 2c). Therefore, various changes in these two positions were investigated *vs* the mannitol acceptor so as to explore possible trends in the fructosylation of this molecule. For this purpose, seven different Ffase Q176 or Q228 variants were independently incubated for 3 h in the presence of 200 g l^−1^ sucrose and 400 g l^−1^ mannitol, and the resultant compounds so generated were quantified (Table 2). In all assays, the main transfer product of the reaction was β-D-fructosyl-D-mannitol, together with 6-kestose, which constituted the major trisaccharide produced by self-transfructosylation of sucrose. Despite the fact that all tested variants produced fructosyl-mannitol, none of them significantly improved the values reached by the wild-type enzyme used as a control, which generated 38.4 g l^−1^ of fructosyl-mannitol and 12.2 g l^−1^ of total FOS, when about 80% of the initial sucrose has been consumed. At best, the Q228E variant produced a slight $\sim$ 8% increase in the level of fructosyl-mannitol, but without substantial change in the ratio fructosyl-mannitol/FOS. Substitutions Q228T and Q228V showed a clear reduction in the hydrolytic activity, and consequently, both of them decreased the total synthesis of fructosylated products (including FOS and fructosyl-mannitol). Neokestose production was not detected using any of the tested Q228 variants and 1-kestose was only observed for the case of Q228N, which again demonstrates the importance of this position in the product specificity of Ffase. On the contrary, every Ffase-Q176 variant assayed so far showed synthesis of 1-kestose, and Q176N even catalyzed the formation of small traces of neokestose; albeit the total amount of FOS was somehow reduced in all three enzymatic versions. The Q176E substitution caused the greatest reduction in the production of FOS ($\sim$ 3.4-fold) without affecting significantly the synthesis of fructosyl-mannitol (37.7 *versus* 38.4 g l^−1^) and increasing the ratio fructosyl-mannitol/FOS by $\sim$ 3.4 times (10.6 *versus* 3.1).

**Table 2:** Concentration (g l^−1^) of the sugar constituents present at maximum fructosyl-mannitol production in reaction mixtures based on sucrose and mannitol with the referred Ffase variants.

Ffase	Sucrose	F-mannitol	Neokestose	1-Kestose	6-Kestose	FOS^†^	Ratio^‡^
*wt*	38.9 ± 2.1	38.4 ± 1.9	1.20 ± 0.06	2.5 ± 0.1	8.5 ± 0.4	12.2	3.1
Q176N	42.9 ± 2.3	16.7 ± 0.9	0.58 ± 0.03	1.82 ± 0.09	7.3 ± 0.4	9.7	1.7
Q176S	60.2 ± 3.1	27.7 ± 1.4	ND	1.50 ± 0.07	5.3 ± 0.2	6.9	4.0
Q176E	36.4 ± 1.9	37.7 ± 1.9	ND	0.90 ± 0.04	2.6 ± 0.1	3.6	10.6
Q228N	27.2 ± 1.4	34.9 ± 1.8	ND	0.80 ± 0.04	12.8 ± 0.7	13.6	2.6
Q228E	22.4 ± 1.2	41.7 ± 2.2	ND	ND	13.8 ± 0.7	13.8	3.0
Q228T	108.2 ± 5.6	17.7 ± 0.9	ND	ND	4.7 ± 0.2	4.7	3.8
Q228V	128.1 ± 6.8	7.9 ± 0.4	ND	ND	4.0 ± 0.2	4.0	2.0

^†^Concentration of FOS: neokestose, 1-kestose, and 6-kestose.

^‡^Ratio between fructosyl-mannitol (F-mannitol) and total FOS.

*ND* Not detected.

Data represents the average of two independent measurements obtained after 3 h of reaction. Standard deviation values are indicated.

## Discussion

Previous work addressed the molecular basis of the existing Ffase transfructosylating mechanism for yielding 6-kestose as the major product, concluding two possible alternative binding modes for the donor sucrose^2,4^. In the first one, sucrose may bind through polar linkages to Asn-254 and Gln-228 at subsite + 1 and + 2, respectively, with the concomitant formation of the β-(2–6)-linked transfructosylation product (Fig. 2d). This arrangement is the preferred location of sucrose to produce 6-kestose. The second binding mode of the donor sucrose maintains the same polar interaction with Gln-228 at subsite + 1, but placing its terminal glucose or fructose unit in a different way stacking against the Trp-76 at subsite + 2, resulting in the synthesis of the secondary products 1-kestose and neokestose. Thereby, the two-fold ratio between 1-kestose and neokestose of the wild-type enzyme^1^ suggests an improved stacking interaction of glucose over fructose at this subsite + 2.

The new study presented here gives novel details of the enzymatic mechanism regulating the transfructosylation ability of Ffase. The complex Ffase-D50A/sucrose showed that the pair Gln-228/Asn-254 is additionally involved in donor substrate recognition by making direct hydrogen bonds with sucrose (Fig. 2b). Therefore, the removal of these side-chains must produce a reduced efficacy in the breakdown of sucrose, which necessarily would result in a smaller amount of the transferred products. In agreement with this, the Q228V/T and N254A mutants showed markedly decreased levels of FOS and fructo-conjugates as a possible consequence of its lower affinity for sucrose donor binding. A similar reduction was observed in the mutant N254E, which might not be able to make an efficient polar linkage to the donor sucrose. Furthermore, and despite Gln-176 not being involved in substrate binding, it participates in the extended network of polar linkages connecting Arg-178 and Gln-228 to the catalytic Glu-230 carboxylate. Accordingly, the changes Q176N and Q176S change the hydrogen bond scheme disturbing the proper arrangement of the active site with the subsequent reduced efficiency in binding and a decreased amount of transfructosylate products. However, the analysis of the other mutants is not as straightforward and suggests a very particular mechanism that do not support our previous assumption that Gln-228 was essentially responsible for conferring affinity to sucrose acceptor, while Asn-254 was mostly responsible of product specificity^4^.

Thus, the N254D replacement reduced $\sim$ 70% the total amount of FOS, while giving a comparable fructosyl-erythritol derivative as the wild-type enzyme. This means that the aspartate side-chain can interact with the alditol acceptor with similar efficiency as asparagine (Fig. 2a) but having a lower affinity to the sucrose acceptor. If the erythritol moiety can establish a polar linkage to the aspartic carboxylate, the same situation would have been expected for the sucrose acceptor but, unexpectedly, this was not the case. The equivalent substitution Q228E gave a similar amount of both FOS and the fructosylated mannitol observed in the wild-type, which could be explained by considering that the glutamate side-chain can make similar polar linkages to the sucrose or mannitol acceptors as glutamine. However, the fact that this mutant only yields 6-kestose indicates that additional effects introduced by the Q/E change must participate to increase the preference towards this trisaccharide. Interestingly, the variant Q176E presented an comparable situation to that previously described in N254D, which had produced an amount of fructosyl-mannitol similar to the wild-type, but reducing the production of FOS by $\sim$ 3 times. In this way, using Q176E variant the ratio between the fructosyl derivative and the FOS was increased to 10.6, a fact that could be an advantage to facilitate the purification of the fructosyl-mannitol.

Finally, the effects observed by the N254T mutation warrant a special consideration. The replacement of asparagine by threonine increased the production of fructosyl-erythritol and the total amount of FOS, which might be explained in terms of a putative new hydrogen bond introduced by the Thr side-chain (in cyan in Fig. 2b) with sucrose. This linkage may have increased the affinity for the binding of sucrose donor in the first step of the reaction. Moreover, Thr-254 will be potentially able to make a polar interaction with Glu-230, thus participating in the network existing in the environment of the acid/base catalyst. This change might also explain a more favorable interaction to sucrose acceptor resulting in a higher amount of FOS and fructosylated erythritol, which in turn could establish a polar linkage to the new Thr-254 in our complex (Fig. 2a). In spite of this, the inverted proportion of 1-kestose/neokestose produced by this mutant is difficult to explain. None of neokestose, 1-kestose, or 6-kestose trisaccharides would be at a proper distance to interact with Asn or Thr at position 254 and, therefore, the stacking interaction between glucose or fructose and Trp-76 should not be affected by this replacement. In contrast, the increased production of blastose obtained by the N254T change is consistent with Thr-254 being able to make a new potential hydrogen bond to the O1 of the fructosyl-β-(2–6)-linked glucose, while the sugar is at the same time stacking to the Trp-314 side-chain (Fig. 2e).

In conclusion, we reported here a detailed structural analysis of the Ffase active site in interaction to fructosyl-alternative acceptors that provides a new piece of information to decipher the sophisticated mechanism regulating the protein specificity. The preference of the Ffase enzyme in producing β-(2–6)-linked FOS was attributed to the presence of several unconserved polar residues near the acid/base catalyst Glu-230 that interact with the sucrose donor substrate to yield 6-kestose as the major product^4^. The results provided in this work showed that, in addition to their ability to make polar linkages to the substrates, these residues take part of an intricate network of polar interactions that seems to be essential, not only to preserve the proper architecture of the active site, but also for maintaining the appropriate electrostatic equilibrium conferring the required charge state of Glu-230 to perform its role in catalysis. Therefore, any change in this environment may alter the network of polar interactions, eventually affecting the acid/base properties of Glu-230, with direct consequences in the specificity of the enzyme. Ffase has been previously shown to be a very useful biocatalyst to obtain fructosylated derivatives and we presented here two point-mutated variants able to improve the produced amount of fructosyl-erythritol (Ffase-N254T) or fructosyl-mannitol (slight in Ffase-Q228E), along with two more (Ffase-N254D/-Q176E) that clearly turn their activity towards the production of these fructosylated polyols to the detriment of the production of FOS. Moreover, the novel details provided here contribute to better depict the molecular basis of the Ffase activity, which will assist further research to expand the range of products with potential biological activity generated by this biocatalyst.

## Materials and methods

### Microorganisms, growth and expression conditions

*Saccharomyces cerevisiae* EUROSCRAF Y02321 (MY4741; *Matα*, *his*3Δ1, *leu*2Δ0, *met*15Δ0, *ura*3Δ0, *YIL162w(SUC2)::kanMX4*) transformants expressing the wild-type β-fructofuranosidase from *Schwanniomyces occidentalis* (Ffase) and the Ffase-D50A (inactive mutant), -Q176N/S/E, -Q228N/T/E/V and -N254D/E/T/A variants were obtained as previously referred^4^. Strains were maintained on SCD agar plates [6.7 g l^−1^ yeast nitrogen base (Difco, USA), 20 g l^−1^ glucose, 0.1 g l^−1^ leucine, 0.05 g l^−1^ histidine, 0.05 g l^−1^ methionine, and 15 g l^−1^ agar] and grown on YPGal [10 g l^−1^ yeast extract, 10 g l^−1^ peptone, and 20 g l^−1^ galactose] for expressing Ffase activity. YPGlu [YPGal but including glucose instead of galactose] was used as a negative expression control medium. Cultures were incubated at 30 ºC using a rotary shaker (230 rpm) and its density followed spectrophotometrically at 660 nm (OD660).

### Purification of Ffase variants from *S. cerevisiae* transformants

Ffase variants expressed in *S. cerevisiae* were purified as described elsewhere^33^. Basically, yeast transformants expressing each of the protein versions were first grown in YPGal medium (1 L) to stationary phase. Then, cultures were filtrated, concentrated using a VivaFlow 50 system (Sartorius AG; Göttingen, Germany), applied to a DEAE-Sephacel chromatography column and proteins eluted using a NaCl linear gradient. Fractions containing apparently pure Ffase were eluted at 0.1 M NaCl, pooled, dialyzed, concentrated, and stored at − 70 °C. All enzymatic purifications were confirmed by SDS-PAGE (9%) gels stained with ProtoBlue Safe Colloidal Coomassie (National Scientific, Atlanta, GA, USA) using standard methods (a representative gel is shown in Supplementary Fig. S4 and S5 online. Protein concentration was estimated by measuring absorbance at 280 nm (NanoDrop spectrophotometer ND-1000).

### Standard β-fructofuranosidase hydrolytic activity assays

Unless otherwise indicated, standard β-fructofuranosidase activity was assayed at 50 ºC for 20 min by measuring the amount of reducing sugars released from 20 g l^−1^ sucrose in 100 mM sodium acetate pH 5.5 using dinitrosalicylic acid (DNS) method adapted to 96-well microplates (50 µl final volume) as previously described^34^. Glucose (0.2—3.0 g l^−1^) was used for the calibration curve. Reaction mixtures without enzyme or sucrose were used as negative controls. One unit of activity (U) was defined as that catalyzing the formation of 1 µmol of reducing sugar per minute under the above-described conditions. Each reaction was performed at least in triplicate*.*

### Ffase-catalyzed transfructosylating reactions

Production of fructosyl-alditols by the Ffase variants was evaluated in 1.5 ml reaction mixtures containing 10 U ml^−1^ of apparently pure enzyme, 200 g l^−1^ sucrose, and either 400 g l^−1^ mannitol or 500 g l^−1^ erythritol in 0.1 M sodium acetate pH 5.5. Blastose synthesis was carried out in similar reactions by replacing alditols with 600 g l^−1^ glucose. Control reactions contained pure enzyme and 200 g l^−1^ sucrose. All mixtures were incubated at 50 ºC for up to 240 min, except for those including 600 g l^−1^ glucose which were maintained for up to 600 min. Samples of 100 µl were withdrawn at different reactions times, boiled for 10 min to inactivate the enzyme, and stored at – 20 ºC. Sugars in the reaction mixtures were analyzed and quantified by HPLC-ELSD.

### HPLC-ELSD analysis

Sugars (and derivatives) in the transfructosylating reactions were identified and quantified by HPLC with a quaternary pump (mod. 600E, Waters Corp.) coupled to a 5-μm Purple-NH_2_ column (4.6 mm × 250 mm) from Análisis Vínicos S.L. (Tomelloso, Spain). An evaporative light-scattering (ELSD) detector (mod. 1000, Polymer Laboratories, Ltd.; Church Stretton, UK) equilibrated at 90 ºC and an automatic injector (mod. 717 Plus, Waters Corp.; Milford, USA) were used. Acetonitrile/water 75:25 (v/v), degassed with an in-line vacuum generator (ser. 200, Perkin-Elmer Corp.; Eden Prairie, USA), was used as a mobile phase at 1.0 ml min^−1^ for a total analysis time of 30 min. The temperature of the column was kept constant at 25 ºC. Obtained data were analyzed using the Empower software (v. 1.0; Waters Corp.). Before injection, each sample was diluted with ultrapure water and passed through a nylon-fiber syringe filter of 0.45-µm-pore size (Scharlau, S.L.; Sentmenat, Spain). All compounds were quantified on the basis of peak area. Each analysis was conducted at least in duplicate. Commercial internal standards were erythritol, mannitol, fructose, glucose, and sucrose from Sigma-Aldrich Corp. (St. Louis, USA); and 1-kestose from TCI-Europe N.V. (Zwijndrecht, Belgium). 6-Kestose, blastose, neokestose, and fructosyl-alditols were synthesized from sucrose using the extracellular β-fructofuranosidases from *Rhodotorula dairenensis*^35^, *Cladosporium cladosporioides*^36^, *Xanthophyllomyces dendrorhous*^37^, and *Sw. occidentalis*^1^, respectively. When standards were not available, the same detector response factor of the most closely related available compound was used.

### Purification and mass spectrometry of the fructosyl derivatives

Biosynthetic reactions were scaled up to 9 ml and the resultant fructosyl-derivatives were purified by semipreparative HPLC in a Waters 600 system equipped with a Sedex 75 ELSD detector (Sedere) using a Kromasil-NH_2_ column (10 mm × 250 mm, 5 μm) as referred^1^. The molecular weight of the putative Fru-Fru-erythritol was assessed by MALDI-TOF mass spectrometry using an Ultraflex III TOF/TOF equipment (Bruker, Billerica, MA, USA) and a neodymium-doped yttrium aluminum garnet (NdYAG) laser. Registers were taken in positive reflector mode within the mass interval 40–5000 Da, with external calibration, and with 10 mg ml^−1^ 2,5-dihydroxybenzoic in methanol:water (9:1) (v/v) as a matrix. Samples (diluted 1:50 in water) were mixed with the matrix in a 20:1 proportion and 0.5 μl were analyzed.

### Crystallization and X-ray structure determination

The inactive mutant Fase-D50A was deglycosylated and crystallized as previously described^4^. Best crystals were grown from 16% PEG 6000, 3% MPD, 0.2 M MgCl_2_, and 0.1 M MES at pH 6.0. To obtain the Ffase-D50/fructosyl-erythritol complex, crystals were transferred for 15 min to a soaking solution containing 19% PEG 6000, 3% MPD, 0.1 M MES at pH 6.0, and 100 mM fructosyl-erythritol; and subsequently into a fresh drop supplemented with 20% glycerol prior to cooling to 100 K in a nitrogen stream. A full data set was collected to 1.88 Å at the XALOC beamline at ALBA (Cerdanyola del Vallés, Spain). Diffraction images were processed with XDS^38^ and merged using AIMLESS^39^ from the CCP4 package^40^. Both complexes were indexed in the P2_1_ space group, with two molecules in the asymmetric unit and 70% solvent content within the unit cell. The structures of the Ffase-D50A complexes were solved by molecular replacement using the atomic coordinates of deglycosylated wild-type Ffase (PDB code: 3KF3) as the search model. Crystallographic refinement was performed using the program REFMAC^41^ within the CCP4 suite with flat bulk-solvent correction and local non-crystallographic symmetry (NCS). Both substrates were fitted manually into the electron density and further refined. At the last stage, water molecules and several N-acetylglucosamine were included, and further model completion was performed with the program Coot^42^ combined with more rounds of positional and individual restrained B-factor refinement. Final refinement parameters for both complexes are reported in the Supplementary Table S1 online. The stereochemistry of the model was checked by using ProCheck^43^ and the figures were generated with PyMOL (The PyMOL Molecular Graphics System, Version 2.0 Schrödlinger, LLC). The atomic coordinates have been deposited in the Protein Data Bank under accession number 6S2B and 6S1T. The transfructosylating FOS products were modelled within the Ffase active site as described previously^2^.

### Automated docking of fructosyl-mannitol into Ffase-D50A

Ffase-D50A coordinates from the fructosyl-erythritol complex were used as a template in order to simulate a real crystallization soaking experiment with fructosyl-mannitol derivative. Fructosyl-mannitol was manually built with PyMOLX11Hybrid (The PyMOL Molecular Graphics System, Version 2.0 Schrödinger, LLC) and AutoDockTools^44^ were used to prepare the coordinates and the ligands to create the .pdbqt files. Polar hydrogens were included and nine amino acids, which are located in the catalytic site, were considered as flexible residues to improve accuracy: Trp-76, Ser-111, Asn-116, Arg-178, Asn-228, Asp-230, Asn-254, Tyr-293 and Trp-314. Furthermore, 18 rotatable torsions were defined in the ligand molecule. A grid box with 30 Å × 40 Å × 40 Å dimensions was defined. AutoDock Vina^45^ default parameters were defined, keeping the exhaustiveness at 8. 20 Solutions were calculated in a binding energy ranked between − 7.2 and − 5.4 kcal/mol. The best solution was selected through chemical and energetic binding criteria. The interactions were evaluated with PyMOL.

### Ethical approval

This article does not contain any trials with human or animal participants performed by any of the authors.

## Supplementary Information

Supplementary Information
