# Supplementary material for: New insights into the molecular mechanism behind mannitol and erythritol fructosylation by β-fructofuranosidase from Schwanniomyces occidentalis
Source: Sci Rep. 2021 Mar 30;11:7158. doi: 10.1038/s41598-021-86568-6 (PMC8010047; doi:10.1038/s41598-021-86568-6)
Supplement: Supplementary file 1 — Supplementary Information [file 41598_2021_86568_MOESM1_ESM.pdf]

New insights into the molecular mechanism behind mannitol and erythritol fructosylation by β-fructofuranosidase from *Schwanniomyces occidentalis*

David Rodrigo-Frutos[1], Elena Jiménez-Ortega[2], David Piedrabuena[1], Mercedes Ramírez-Escudero[2], Noa Míguez[3], Francisco J. Plou[3], Julia Sanz-Aparicio[2*], & María Fernández-Lobato[1*]

[1]Centro de Biología Molecular Severo Ochoa (CBMSO; UAM-CSIC), Departamento de Biología Molecular, Facultad de Ciencias, Universidad Autónoma de Madrid, Nicolás Cabrera 1, Madrid, 28049, Spain.

[2]Instituto de Física-Química Rocasolano (CSIC), Departamento de Cristalografía y Biología Estructural, Serrano 119, Madrid, 28006, Spain.

[3]Instituto de Catálisis y Petroleoquímica (ICP-CSIC), Marie Curie 2, Madrid, 28049, Spain

[*]Corresponding authors: J. Sanz-Aparicio (xjulia@iqfr.csic.es); M. Fernández-Lobato (mfernandez@cbm.csic.es)

-Table S1. Crystallographic statistics.
-Figure S1. Schematic view of the reactions catalyzed by Ffase on sucrose.
-Figure S2. HPLC chromatograms from transfructosylating reactions produced by N254T (a) or wild-type (b) Ffase variants.
-Figure S3. Mass spectrum of the reaction mixture obtained with the Ffase-N254T variant in reactions containing sucrose and erythritol.
-Figure S4. SDS-PAGE analysis of the purified Ffase variants.
-Figure S5. The full-length gel of the Figure S4 is presented.

Table S1. Crystallographic statistics.

**Crystal data**	Ffase-D50A / Fru-erythritol	Ffase-D50A / Sucrose
Space group	P 2_1	P 2_1
Unit cell parameters		
a (Å)	60.48	60.90
b (Å)	92.54	93.20
c (Å)	116.11	116.61
β (º)	104.71	104.90
**Data collection**		
Beamline	XALOC (ALBA)	XALOC (ALBA)
Temperature (K)	100	100
Wavelength (Å)	0.97926	0.97926
Resolution (Å)	49.33-1.88 (1.91-1.88)	49.69-2.09 (2.13-2.09)
**Data processing**		
Total reflections	536923 (25965)	369570 (22936)
Unique reflections	98,961 (4791)	74544 (4621)
Multiplicity	5.4 (5.4)	5.0 (5.0)
Completeness (%)	98.6 (97.9)	99.9 (99.8)
Mean I/σ (I)	9.2 (3.0)	8.6 (2.5)
R_{merge}† (%)	12.3 (63.0)	13.8 (56.9)
R_{pim}†† (%)	5.9 (30.0)	7.0 (28.6)
Molecules per ASU	2	2
**Refinement**		
R_{work} / R_{free}††† (%)	15.39 /17.85	17.49/21.36
**Nº of atoms/average B** ($Å^2$)		
Protein	8341/19.21	8308/20.28
Carbohydrate	226/37.91	248/37.18
Water Molecules	849/30.72	794/27.06
All atoms	9416/20.69	9350/21.30
**Ramachandran plot** (%)		
Favoured	96	96
Outliers	0	0
**RMS deviations**		
Bonds (Å)	0.0072	0.0065
Angles (°)	1.4496	1.4589
**PDB accession codes**	6S2B	6S1T

Values in parentheses are for the high resolution shell

†$R_{merge} = \sum_{hkl} \sum_i | I_i(hkl) – [I(hkl)]| / \sum_{hkl} \sum_i I_i(hkl)$, where $I_i(hkl)$ is the ith measurement of reflection hkl and [I(hkl)] is the weighted mean of all measurements.

††$R_{pim} = \sum_{hkl} [1/(N - 1)]\ 1/2 \sum_i | I_i(hkl) – [I(hkl)]| / \sum_{hkl} \sum_i I_i(hkl)$, where N is the redundancy for the hkl reflection.

†††R_{work} / $R_{free} = \sum_{hkl} | Fo – Fc | / \sum_{hkl} | Fo |$, where Fc is the calculated and Fo is the observed structure factor amplitude of reflection hkl for the working / free (5%) set, respectively.

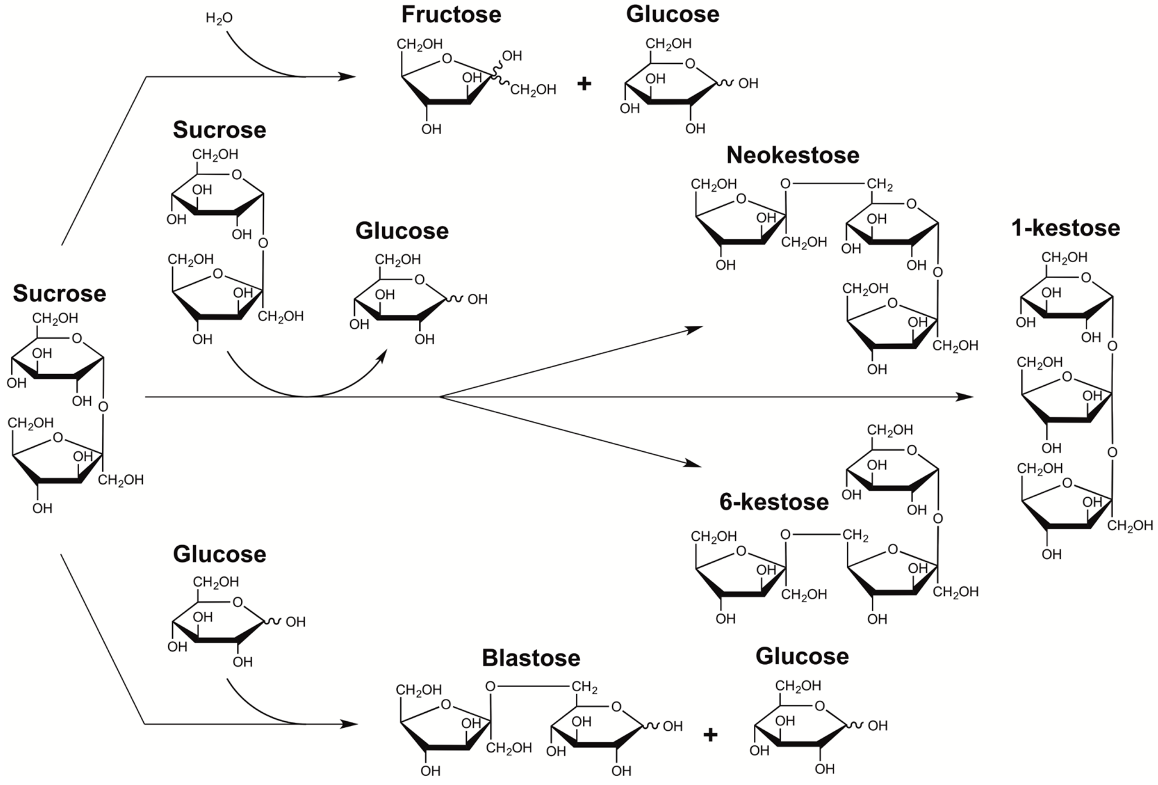

Figure S1. Schematic view of the reactions catalyzed by Ffase on sucrose. This enzyme cleaves the β-(2→1) linkage of a donor sucrose unit (hydrolytic activity) and transfers the fructosyl moiety into an acceptor sucrose or glucose unit (transferase activity) generating: blastose [β-D-Fru-(2→6)-Glc], neokestose [β-D-Fru-(2→6)-α-D-Glc-(1→2)-β-D-Fru], 1-kestose [β-D-Fru-(2→1)-β-D-Fru-(2→1)-α-D-Glc] and 6-kestose [β-D-Fru-(2→6)-β-D-Fru-(2→1)-α-D-Glc].

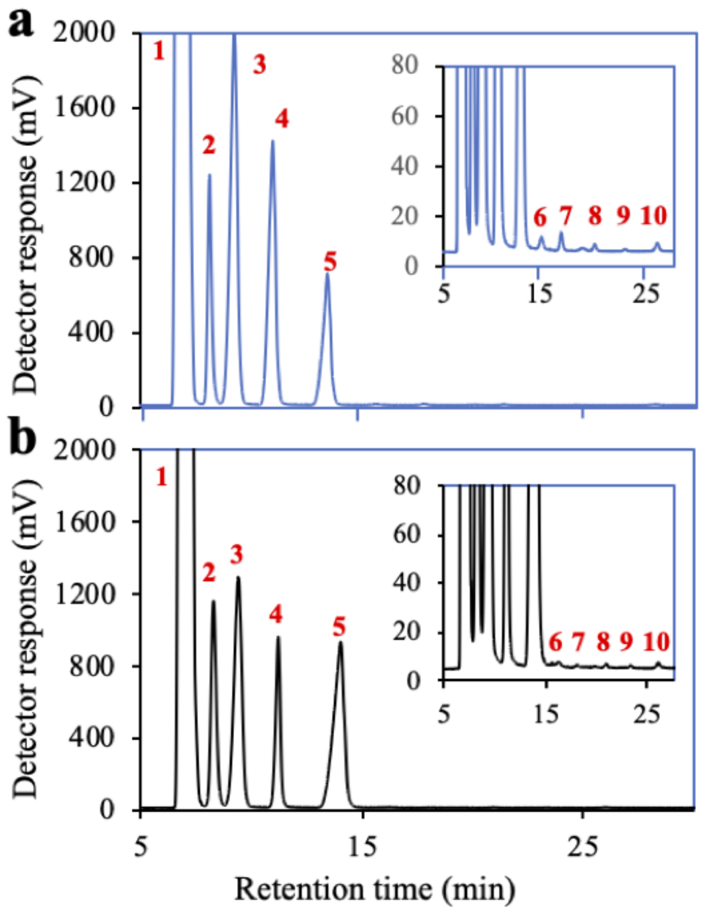

Figure S2. HPLC chromatograms from transfructosylating reactions produced by N254T (a) or wild type (b) Ffase variants. 10 U ml^{-1} of pure enzyme was incubated in 200 g l^{-1} sucrose and 500 g l-1 erythritol. Analyses were conducted at the point of maximum fructosyl-erythritol production. Upper right corners show a close-up view of the corresponding figures including the detector response range. Peaks assignations: (1) erythritol, (2) fructose, (3) glucose, (4) fructosyl-erythritol, (5) sucrose, (6) blastose, (7) potential (fructosyl)$_2$-erythritol, (8) neokestose, (9) 1-kestose, and (10) 6-kestose.

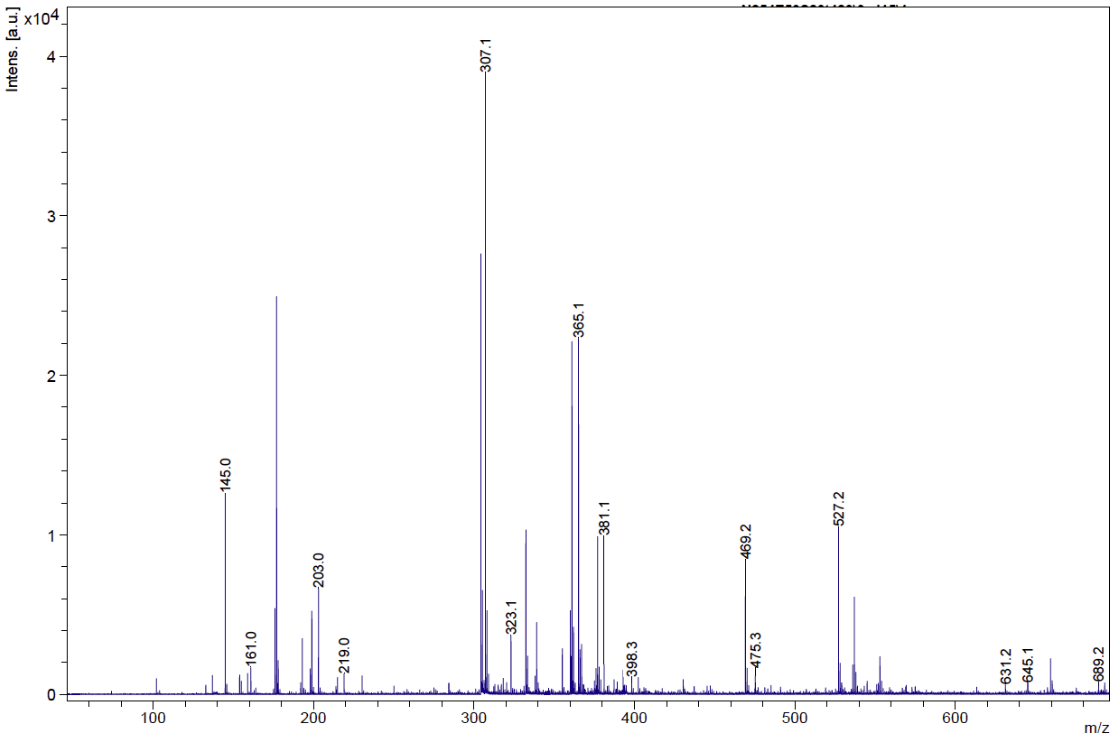

Figure S3. Mass spectrum of the reaction mixture obtained with the Ffase-N254T variant in reactions containing sucrose and erythritol. Reaction conditions: 1.5 ml mixtures containing 200 g l^{-1} sucrose and 500 g l^{-1} erythritol in 0.1 M sodium acetate at pH 5.5 were incubated at 50 ºC for 240 min. Molecular masses of chemical constituents plus sodium ion (~ 23 u) were detected in positive mode. Peaks of $[M+Na]^+$ m/z of 307.1, 469.2, and 631.2 were assigned respectively as fructosyl-erythritol, potential (fructosyl)$_2$-erythritol, and negligible traces of hypothetical (fructosyl)$_3$-erythritol. Peaks of $[M + Na]^+$ m/z of 145.0 and 365.1 correspond to erythritol (substrate) and the disaccharides sucrose (substrate) or blastose (product), respectively. Peaks of 203.0, 527.2, and 689.2 to monosaccharides (fructose, glucose), trisaccharides (1-kestose, 6-kestose, neokestose), and minimal quantities of unidentified tetrasaccharides, respectively. Peaks of $[M + K]^+$ m/z of 161.0, 219.0, 323.1 and 381.1, corresponding to erythritol, monosaccharides, fructosyl-erythritol and disaccharides, including K^+ (~ 39 u) instead of Na^+ were also assigned, whereas others such as those between 161.1 and 203.0 could not be assigned.

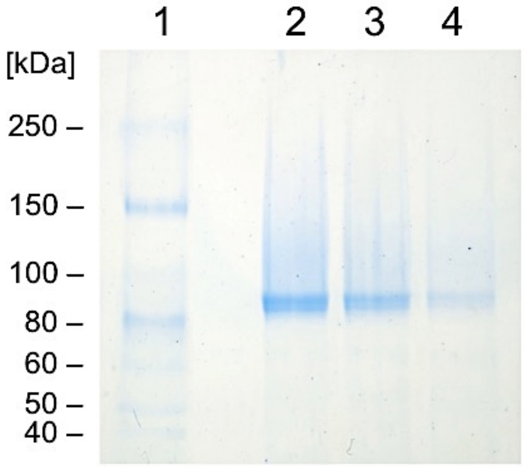

Figure S4. SDS-PAGE analysis of the purified Ffase variants. Lane 1: Unstained protein standards ranging 10-250 KDa (New England Biolabs Inc.; Ipswich, USA) used for molecular weight estimations. Lane 2-4: 0.5 µl of purified enzyme Ffase-D50A, -N254T, and -Q228E; respectively. Numbers at the left indicate the positions of molecular mass standards in KDa.

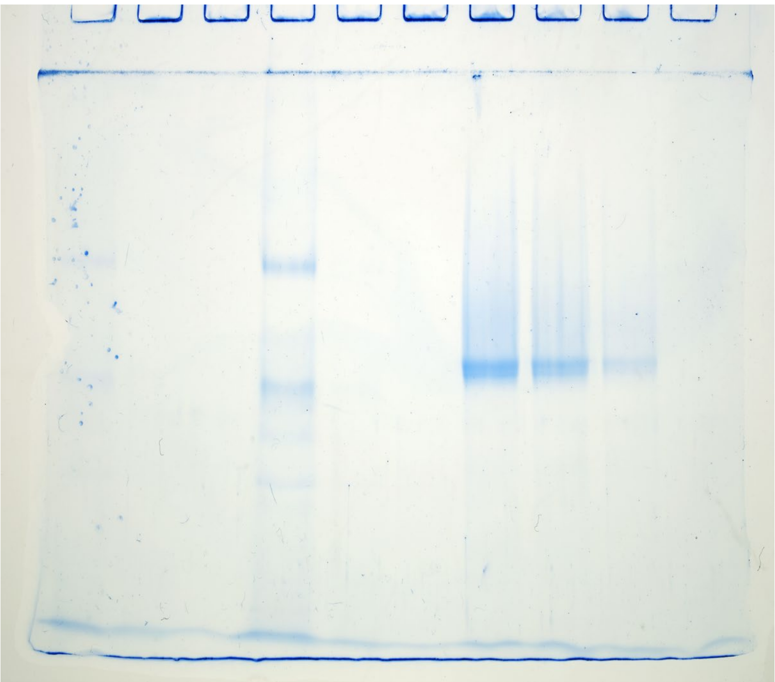

Figure S5. The full-length gel of the Figure S4 is presented.
